# Anaemia and its association with month and blood phenotype in blood donors in Fako division, Cameroon

**DOI:** 10.1186/s12878-016-0070-8

**Published:** 2016-11-28

**Authors:** Tebit Emmanuel Kwenti, Tayong Dizzle Bita Kwenti

**Affiliations:** 1 Regional Hospital Buea, P.O. Box 32, Buea, South West Region Cameroon; 2 Department of Medical laboratory Sciences, Faculty of Health Sciences, University of Buea, P.O. Box 63, Buea, Cameroon; 3 Department of Microbiology and Parasitology, Faculty of Science, University of Buea, P.O. Box 63, Buea, Cameroon

**Keywords:** Anaemia, Blood donors, Prevalence, Association, Blood phenotype, Season, Fako Division, Cameroon

## Abstract

**Background:**

Anaemia is one of the main factors in the deferral (disqualification) of blood donors following haematological screening. There is paucity of data on the prevalence of anaemia in blood donors in Sub-Saharan Africa. This study was undertaken to determine the prevalence of anaemia and its association with month and blood phenotype in blood donors in Fako division of Cameroon.

**Methods:**

Blood donors were recruited between the 1st of January and 31st of December 2014, and their haemoglobin concentration (Hb) was determined using a haemoglobinometer. Anaemia was considered as Hb < 12 g/dl for females and Hb < 13 g/dl for males. The ABO and Rhesus blood groups were determined using standard techniques with monoclonal antibodies and the Coombs’ test. The Pearson’s chi-square, Pearson’s correlation, student T test, ANOVA, univariate and multivariable logistic regression analyses adjusting for gender and age as categorical variable were all performed as part of the statistical analysis.

**Results:**

A total of 1896 blood donors predominantly males (91.35%) took part in the study. The mean age of the donors was 32 ± 7.81 years. On average, donors had donated blood 5.07 ± 3.54 times in their lifetime. The prevalence of anaemia observed in this study was 31.44% (95% CI: 29.35–33.58). The prevalence of anaemia was higher in females (*p* ≤ 0.0001) and in participants of age 20 years and below (*p* = 0.001). A marginal association was observed between prevalence of anaemia and season (*p* = 0.051). Furthermore, a significant association was observed between prevalence of anaemia and the blood group AB (*p* = 0.001). The risk of developing anaemia was higher in females compared to males (OR = 2.7, *p* < 0.0001). The mean Hb observed in this study was 13.42 ± 1.65; the mean Hb was not observed to be associated with the month or season adjusting for age and gender.

**Conclusion:**

This study revealed a high prevalence of anaemia which translates to a high rate of donor deferral as a result of anaemia in the study area. The prevalence of anaemia was observed to be associated with the blood phenotype and the month, but not the season (dry or rainy). Further studies will be needed to ascertain the aetiology and associated factors for anaemia in blood donors in the study area.

## Background

Anaemia is a condition where there is a decrease in the amount of red blood cells or less than the normal quantity of haemoglobin in the blood. It is the most common disorder of the blood and it symbolizes both poor nutrition and poor health [1]. The causes of anaemia are multifactorial and may result from blood loss, decreased red blood cell production or increased red blood cell breakdown [2]. Anaemia is a global public health problem affecting nearly a quarter of the world’s population [3]. Although its effect is felt in both developing and developed countries, developing countries are the most affected [4]. The management of anaemia commonly involves the use of iron pills, intravenous iron, and erythropoiesis-stimulating medications or by blood transfusion on a case-by-case basis.

The impact of anaemia on maternal and child health is well recognised. Severe anaemia has been linked to increased risk of maternal and child mortality [1, 5, 6]. Anaemia has also been linked to impaired psychological and physical development, behaviour, and work performance of the population [7]. In Cameroon, like most countries in Sub-Saharan Africa, the prevalence of anaemia is very high. In 2011, the prevalence was estimated at 63.3% in children and 49.5% in pregnant women [8]. Malnutrition is one of the main culprit for anaemia in Africa [9]. In addition to malnutrition, there are also infectious causes of anaemia in Cameroon including malaria and intestinal parasitic infections [10–12].

The impact of anaemia on a country’s blood banking services has received relatively little attention. Anaemia is one of the main reasons for deferral (disqualification) of potential blood donors [13]. In countries where the prevalence of anaemia is high, this will have a profound effect on the blood stock, eventually compromising the quality of health care. This is not very unusual in developing countries like Cameroon where shortages of blood and blood products are frequent due to the high demand driven by the high rate of anaemia.

The prevalence of anaemia doesn’t seem to be constant all year round. In one study, Bondevik et al. [14] had observed a clear seasonal variation of the risk of anaemia in pregnant Nepali women, associated with rainfall and temperature. An earlier study by Palva and Salokannel [15] had also reported a seasonal variation in megaloblastic anaemia. Studies on the seasonal variation of anaemia among blood donor are not readily available.

In Cameroon, data on the prevalence of anaemia among blood donors is lacking in the scientific literature. It was against this drawback that we conducted this study to determine the prevalence of anaemia among blood donors in the Fako Division of Cameroon. The association between anaemia and blood phenotype, and between anaemia and month were also determined. This will generate baseline data which hopefully will be useful in the planning and implementation of blood transfusion services in the country.

## Methods

### Study design and duration

This was a cross-sectional study which started on the 1st of January and concluded on the 31st of December 2014. The study participants were donors who came to donate blood in the Regional Hospital of Buea during the study period.

### Study area

The study was performed in Fako Division (4°10'00''N, 9°10'00''E) of the South West Region of Cameroon. The department covers an area of 2093 km^2^ with an elevation of 2707 m above sea level. Fako Division has an estimated population of 534,854 [16]. The major towns in Fako Division include Buea, Idenau, Limbe (Divisional headquarter), Muyuka and Tiko. The climate of Fako division is generally hot and dry with the exception of Buea where the climate tends to be humid because of its location at the foot of Mount Cameroon. Fako division has two seasons, the rainy and the dry seasons. The rainy season is usually between April and September, and the dry season is between October and March.

### Study population

Blood donors (males or females) in the Regional Hospital of Buea were approached to take part in the study. Participants were to be between the ages 17 and 52 years and of weight ≥ 50 kg. All participants were expected to provide a signed informed consent which was duly explained to them in English, French or the local broken English language. Excluded from the study were individuals who did not meet the criteria for blood donation (unfit) such as donors on any medication, women who were breastfeeding or those on menstruation, and donors who had donated blood within 3 months prior to the study.

Participants were consecutively recruited into the study as they came to donate. Participants were enrolled just once and a single blood sample was collected from the participants.

### Measurement of blood haemoglobin concentration (Hb)

Participants’ Hb was measured using a haemoglobinometer (ACON Mission Plus Hb, ACON Laboratories, Inc., USA). Briefly, about 10 μl of capillary blood from a finger prick was placed on the sample pad on the test strip in the haemoglobinometer using a capillary tube and the haemoglobin concentration (Hb) was read within 20 s. The haemoglobinometer was recalibrated on a daily bases using the control strip supplied by the manufacturer. In this study, anaemia was defined according to the WHO definition of Hb level lower than 12.0 g/dl for females and 13.0 g/dl for males [4]. The severity of anaemia was classified based on the WHO scheme as mild (Hb ≥ 11 but less than normal), moderate (Hb between 8 and 10.9 g/dl) and severe (Hb < 8) [17].

### Blood group determination

The ABO and Rhesus (D) blood groups were determined using a commercially available kit for blood grouping (HUMAN DIAGNOSTIC, Germany). In determining ABO and Rhesus blood group, 4 spots of 30 μl of washed red cells were placed on a clean plate and anti-A, anti-B, anti-AB and anti-D grouping sera were added, mixed and rocked for 2mins on a mixer. Positive results were shown by haemagglutination.

Reverse ABO blood grouping was performed to confirm the blood groups of the participants by determining the reaction of the participants’ serum to known ABO washed red cells.

### Statistical analysis

Statistical analysis was performed using Stata® version 12.1 (StataCorp LP) statistical package. Statistical tests performed included univariate and multivariable logistic regression analyses adjusting for gender and age as categorical variable and the Pearson’s chi-square for qualitative variables, the student T test, ANOVA, and Pearson’s correlation analysis for quantitative variables. All quantitative variables were normally distributed. Statistical significance was set at *p* < 0.05.

## Results

By the end of the study, 1896 potential blood donors were enrolled. Among them were 1,732 (91.35%) males and 164 (8.65%) females. The participants were between 17 and 52 years of age (mean ± SD = 32 ± 7.81). The donors had donated blood on the average 5.07 ± 3.54 times (range: 1–19) in their lifetime.

Five hundred and ninety six (596) of the 1896 donors were anaemic giving a prevalence of 31.44% (95% CI: 29.35–33.58). The prevalence of anaemia was higher in females 87/164 (53.1%) compared to males 509/1732 (29.4%). A significant association was observed between anaemia and gender (χ^2^ = 38.91, *p* ≤ 0.0001). Overall, the prevalence of anaemia was highest in participants of age 20 years and below (62.5%), and lowest in participants between 30 and 39 years of age (28.6%) (See Table 1). A significant association was observed between prevalence of anaemia and age (χ^2^ = 17.32, *p* = 0.001) (See Table 1). The association between prevalence of anaemia and age was significant among males (χ^2^ = 15.369, *p* = 0.002) but not among females (χ^2^ = 2.788, 0.424) (See Table 1).

**Table 1:** Prevalence of anaemia stratified according to age

Age category	Males		Females		Total	
	N	Anaemic (%)	N	Anaemic (%)	N	Anaemic (%)
≤20	25	15 (60.0)	7	5 (71.4)	32	20 (62.5)
21–29	702	200 (28.5)	93	51 (54.8)	795	251 (31.6)
30–39	611	165 (27.0)	39	21 (53.9)	650	186 (28.6)
≥40	394	129 (32.7)	25	10 (40.0)	419	139 (33.2)
Total	1732	509 (29.4)	164	87 (53.1)	1896	596 (31.4)

Overall, the prevalence of anaemia was highest in the month of February (39.7%) and lowest in October (25.2%) (See Table 2). A marginal association was observed between the prevalence of anaemia and month (χ^2^ = 19.62, *p* = 0.051). Among females, the prevalence was highest in March (66.7%) and lowest in July (33.3%), while among males, the prevalence was highest in February (39.5%) and lowest in October (20%) (See Table 2).

**Table 2:** Prevalence of anaemia stratified according to month and gender in the study population

Month	Males		Females		Total		Univariate analysis	Multivariate analysis
	N	Anaemic (%)	N	Anaemic (%)	N	Anaemic (%)	OR (95% CI)	OR (95% CI)
Jan	120	32(26.7)	8	3(37.5)	128	35(27.3)	1.00	0.91 (0.55–1.51), *p* = 0.716
Feb	124	49(39.5)	12	5(41.7)	136	54(39.7)	1.75 (1.04–2.94), *p* = 0.038	1.54 (0.96–2.47), *p* = 0.077
Mar	133	33(24.8)	6	4(66.7)	139	37(26.6)	0.96 (0.56–1.66), *p* = 0.894	0.90 (0.55–1.48), *p* = 0.677
Apr	172	46(26.7)	20	10(50.0)	192	56(29.2)	1.04 (0.67–1.8), *p* = 0.723	0.92 (0.59–1.45), *p* = 0.729
May	161	53(32.9)	12	3(25.0)	173	56(32.4)	1.16 (0.7–1.9), *p* = 0.548	1.13 (0.72–1.79), *p* = 0.591
Jun	113	36(31.9)	10	5(50.0)	123	41(33.3)	1.33 (0.77–2.28), *p* = 0.302	1.16 (0.71–1.91), *p* = 0.555
Jul	111	32(28.8)	6	2(33.3)	117	34(29.1)	1.09 (0.62–1.9), *P* = 0.765	1.01 (0.6–1.69). *p* = 0.973
Aug	119	31(26.1)	9	5(55.6)	128	36(28.1)	1.04 (0.6–1.8), *p* = 0.889	0.91 (0.55–1.50), *p* = 0.700
Sep	155	43(27.7)	22	12(54.6)	177	55(31.1)	1.2 (0.73–1.98), *p* = 0.48	1.00
Oct	160	32(20.0)	20	11(55.0)	180	43(25.2)	0.83 (0.5–1.4), *p* = 0.492	0.71 (0.44–1.14), *p* = 0.154
Nov	233	73(31.3)	28	20(71.4)	261	93(35.6)	1.47 (0.93–2.34), *p* = 0.102	1.29 (0.85–1.96), *p* = 0.226
Dec	131	49(37.4)	11	7(63.6)	142	56(39.4)	1.73 (1.04–2.89), *p* = 0.036	1.54 (0.96–2.47), *p* = 0.071
Total	1732	509(29.39)	164	87(53.05)	1896	596(31.44)		

The prevalence of anaemia in the Rainy season was 32.3% (318/986) and in the Dry season, it was 30.6% (278/910). No significant association was observed in the prevalence of anaemia with season (χ^2^ = 0.64, *p* = 0.425).

The risk of becoming anaemic was higher in females compared to males [OR = 2.7 (95% CI: 1.96–3.75), *p* ≤ 0.0001]. Univariate analysis (with January as the reference month) revealed that the risk of becoming anaemic was higher in the month of February (OR = 1.75, *p* = 0.038) and December (OR = 1.73, *p* = 0.036) (see Table 2). Furthermore univariate analysis (with February as the reference month) revealed that the risk of becoming anaemic was lower in the months of January (OR = 0.57, *p* = 0.038), March (OR = 0.55, *p* = 0.029) and October (OR = 0.48, *p* = 0.003). However multivariate analysis adjusting for age and gender revealed that the risk of becoming anaemic was comparable across the different months of the year (see Table 2).

In all, 457 (76.7%) of the participants had mild anaemia, 129 (21.6%) had moderate anaemia and 10 (1.7%) had severe anaemia (see Table 3). No significant association was observed between the degree of anaemia and month (χ^2^ = 27.26 *p* = 0.202) or season (χ^2^ = 2.23 *p* = 0.328).

**Table 3:** Severity of anaemia with respect to month in the study population

Month	Degree of anaemia			Total
	Mild (%)	Moderate (%)	Severe (%)	
Jan	30(85.7)	5(14.3)	0(0)	35
Feb	45(83.3)	9(16.7)	0(0)	54
Mar	30(81.1)	6(16.2)	1(2.7)	37
Apr	44(78.6)	12(21.4)	0(0)	56
May	50(89.3)	6(10.7)	0(0)	56
Jun	31(75.6)	10(24.4)	0(0)	41
Jul	29(85.3)	5(14.7)	0(0)	34
Aug	23(63.9)	12(33.3)	1(2.8)	36
Sep	39(70.9)	15(27.3)	1(1.8)	55
Oct	34(79)	7(16.3)	2(4.7)	43
Nov	63(67.7)	27(29)	3(3.2)	93
Dec	39(69.6)	15(26.8)	2(3.6)	56
Total	457(76.7)	129(21.6)	10(1.7)	596

**Fig. 1:**
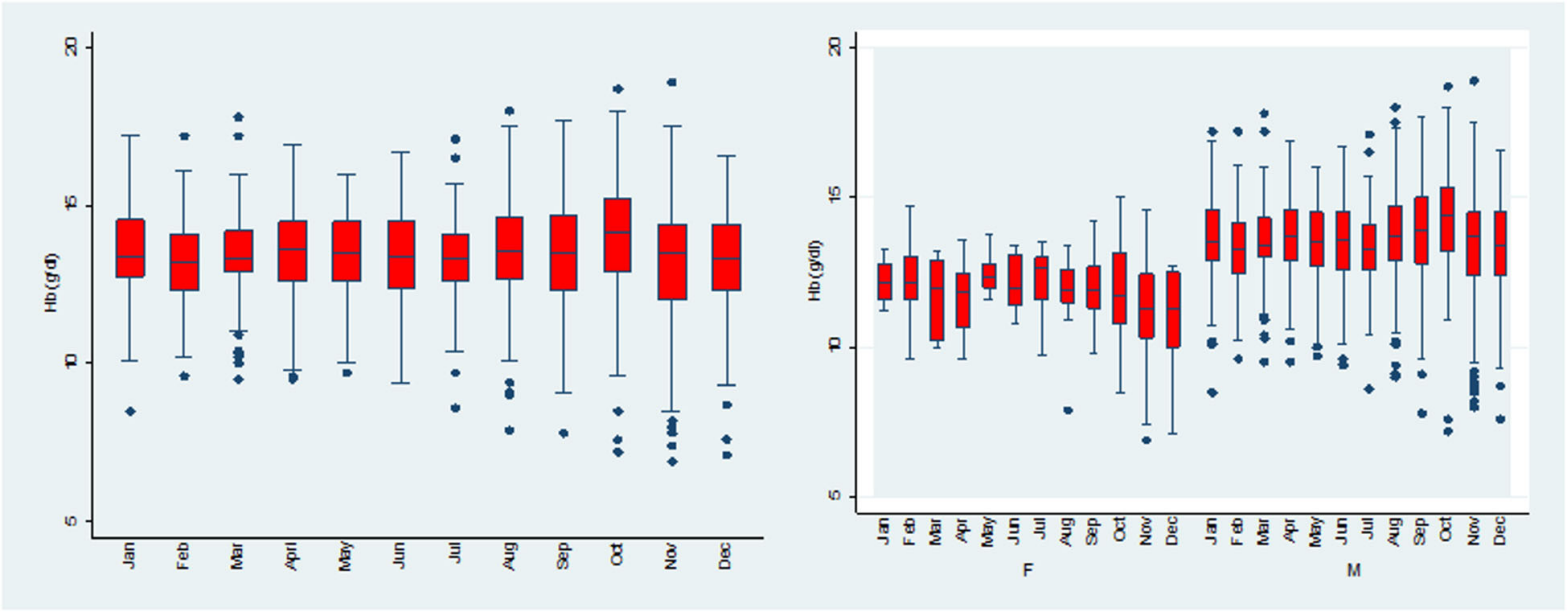
Monthly distribution of Hb in the study population overall (*left*), and stratified according to gender (*right*). No significant association was observed between Hb and month adjusting for age and gender

Overall, the mean Hb in the study population was 13.42 ± 1.65 (range: 6.9–18.9). The mean Hb in females was 11.78 g/dl ± 1.44 (range: 6.9–15), while it was 13.57 ± 1.59 (range: 7.2–18.9) in males. The mean Hb was highest in the month of October and lowest in December (see Fig. 1). In males the mean Hb was highest in October, meanwhile in females, it was highest in May. In males, the mean Hb was lowest in February while in females, it was lowest in December (see Fig. 1). Multivariate analysis adjusting for gender and age revealed no significant association between mean Hb and month (*p* = 0.079).

**Fig. 2:**
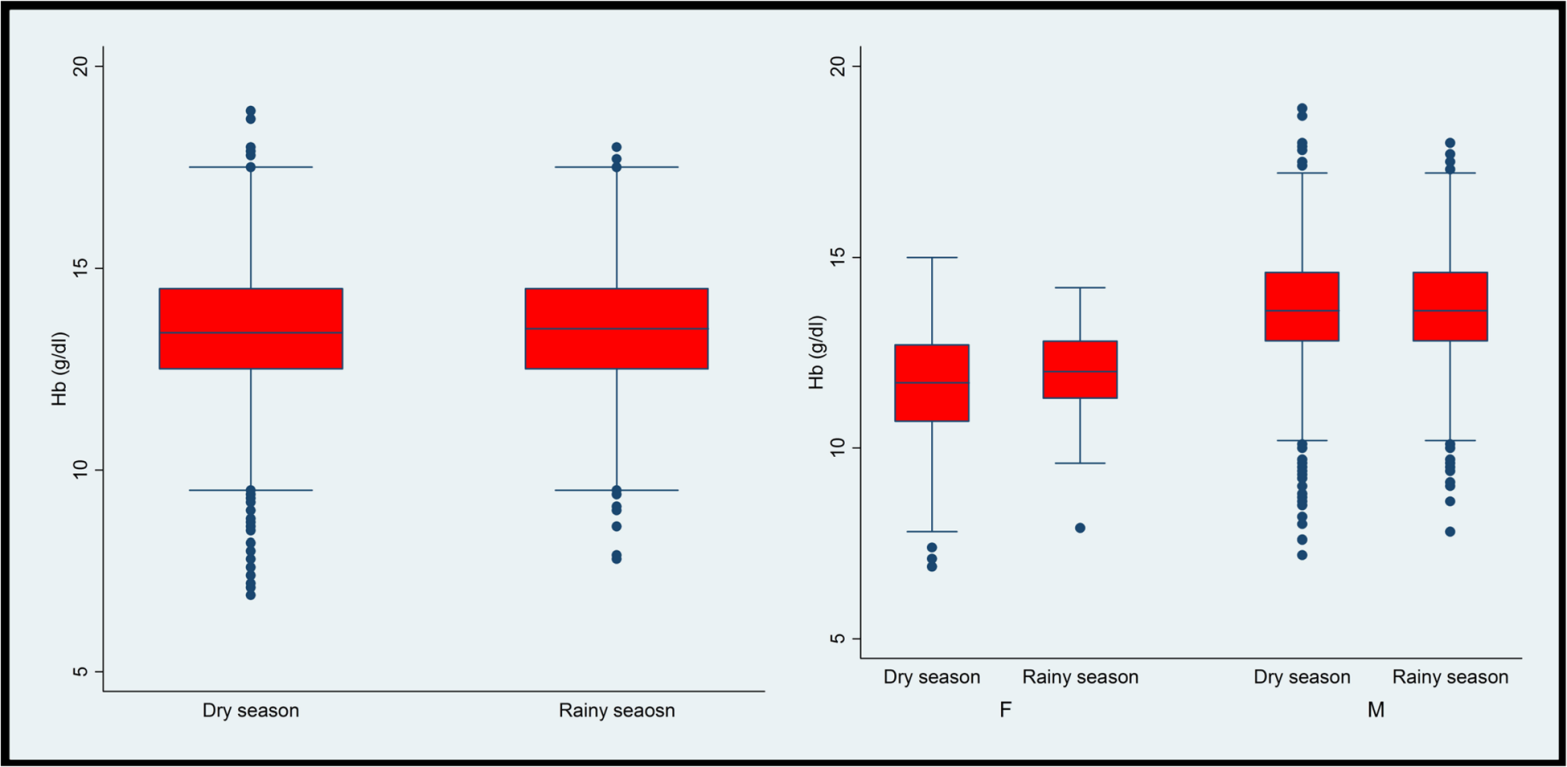
Distribution of mean Hb according to season in the study population. The mean Hb was observed to be higher in the rainy season overall (*left*) and in both males and females (*right*). The difference in the mean Hb between the two seasons was not observed to be significant (*p* = 0.350) adjusting for age and gender

In this study, no significant difference was observed in the mean Hb between the rainy and the dry season (*p* = 0.350) adjusting for age and gender (see Fig. 2).

In the current study, no significant correlation was observed between Hb and age (*r* = 0.043, *p* = 0.062).

The prevalence of anaemia was highest in participants with blood group AB 16(64%) (see Table 4). A significant association was observed between anaemia and blood group (χ^2^ = 15.63, *p* = 0.001).

**Table 4:** The distribution of anaemia with respect to blood phenotype

Blood group	N	Anaemic (%)	χ^2^	*P*-value
A	231	73(31.6)	15.63	0.001
AB	25	16(64)		
B	216	78(36.1)		
O	1424	429(30.1)		
Total	1896	596		

In all, 1827 (96.4%) of the participants were Rhesus (D) positive while 69 (3.6%) were Rhesus (D) negative. The prevalence of anaemia was higher among participants that were Rhesus (D) positive 575 (31.5%) compared to those that were Rhesus (D) negative 21 (30.4%). However no significant difference was observed between prevalence of anaemia and the Rhesus (D) antigen (χ^2^ = 0.033, *p* = 0.855).

## Discussion

Anaemia is one of the main factors of clinical deferral of blood donors in developing countries including Cameroon. In this study, we investigated the prevalence of anaemia and its association with blood phenotypes as well as month among blood donors in Fako division of Cameroon. A high prevalence of 31.44% was observed in this study. The prevalence was higher compared to the 16% reported in Nigeria [18], 4.2% in Brazil [13], and 1.8% in India [19]. The difference between the prevalence of anaemia in these studies compared to ours could be attributed to the differences in the nutritional habits of these populations. Malnutrition and infectious diseases (such as malaria, intestinal parasitic infections, HIV) are some of the main factors contributing to the high prevalence of anaemia especially in children in Cameroon [12]. The high prevalence of anaemia implies 31.44% of the donors were deferred as a result of the anaemia over the year thereby constituting a huge loss of blood stock. Low supply of blood in a country with a high prevalence of anaemia like Cameroon will compromise the blood supply and therefore have an adverse effect on the quality of health care. In this study, the prevalence of anaemia was significantly higher in females compared to males, which is in line with studies performed elsewhere [19, 20]. Females were also observed to be at increased risk of anaemia in comparison to their male counterparts (OR = 2.7, *p* ≤ 0.0001). This is not unusual owing to the fact that women often fail to compensate their menstruational blood loss. In this study, 8.65% of the donors were females. Pregnancy, lactation and menstruation are some of the causes preventing blood donation in females. This may have resulted in a selection bias against females consequently influencing the true representation of the prevalence of anaemia in this group. The finding of fewer female donors in this study corroborate with work done by Silva et al. [13]. In this study, the prevalence of anaemia was observed to be higher in participants of 20 years and below irrespective of gender. A significant association was observed between prevalence of anaemia and age (*p* = 0.001). The finding of higher prevalence of anaemia among younger donors could be attributed to the fact that many of them were students who undergo a lot of stress with their study and hardly feed well. The association between prevalence of anaemia and age was significant among males but not among females. This difference could be due to the observation that the prevalence of anaemia was generally high among females irrespective of the age.

In this study, it was observed that the donors had donated blood on the average of 5 times in their lifetime. Studies have shown that there is an increasing depletion of the iron stores with increasing number of blood donation [21–23]. The high frequency of blood donation observed in this study could have also contributed to the high prevalence of anaemia observed in this study.

We also observed the prevalence of anaemia to be highest in February and lowest in October. In the study area, the dry season is usually between October and March meanwhile the rainy season is between April and September. February which has the highest rate of anaemia falls in the heart of the dry season during which there is scarcity of foodstuffs including vegetables, which are also very expensive where available. This negatively affects the feeding habit of the population of Fako hence the high rate of anaemia. October which has the lowest prevalence of anaemia falls in the transition between the dry and the rainy season. During this period there is abundance of foodstuffs especially vegetables, which are also cheaper. At this time of the year, vegetables constitute a large part of the diet hence the lower prevalence of anaemia. A marginal association was observed between prevalence of anaemia and months in this study. The prevalence of anaemia was higher in the dry season compared to the rainy season which is not unusual. However no significant association was observed between prevalence of anaemia and season. The risk of developing anaemia was also observed to be higher in February and December but multivariate analysis adjusting for age and gender revealed similar risk of anaemia across the different months of the year. Like the prevalence of anaemia, no significant association was observed between the degree of anaemia and month.

The mean Hb observed in this study was 13.42 g/dl and ranged between 6.9 and 18.9 g/dl. The mean Hb was lower in females (11.78 g/dl) compared to males (13.57 g/dl). This is not unusual owing to the fact that the Hb of females is generally always lower than the Hb in males for reasons highlighted above. The mean Hb was highest in October and lowest in December. In this study, no significant association was observed between Hb and month (*p* = 0.079) adjusting for age and gender. The variation in the mean Hb with month could be attributed to the same factors associated with the prevalence of anaemia highlighted above. In females, the mean Hb was highest in the month of May and lowest in December, which is contrary to males in which mean Hb was observed to be highest in October and lowest in February. Like with the prevalence of anaemia in this study, no significant association was observed between Hb and season (*p* = 0.350), neither was there any significant association between Hb and age (*p* = 0.062).

In this study, it was observed that anaemia was significantly associated with blood group AB (*p* = 0.001). Not many studies have associated a particular blood group with anaemia in the literature. A previous study in 1956 had linked pernicious anaemia to blood group A [24]. In this study we did not determine the aetiology of anaemia which prevented us from knowing the exact type of anaemia associated with blood group AB. The prevalence of anaemia was higher in donors who were Rhesus (D) positive (31.5%) compared to Rhesus (D) negative (30.4%) donors. However this difference was not observed to be significant (*p* = 0.855). This difference could be attributed to the fact that the majority of donors were Rhesus (D) positive (96.4%).

In the current study, we did not determine the aetiology and risk factors for anaemia in the study population and this constitutes a major limitation. Studies are therefore required to ascertain the aetiology of anaemia in the study population as well as make prepositions to reduce the high rate of anaemia in blood donors in the study area.

## Conclusion

A high prevalence of anaemia (31.44%) was observed which translates to an equivalent number of blood donors deferred as a result of anaemia in the study timeframe. The prevalence of anaemia was significantly higher in females compared to males and in participants of age 20 years and below. A marginal association was observed between the prevalence of anaemia and month but not with the season. The risk of developing anaemia was comparable across the different months of the year. Female blood donors and donors with the blood group AB were the most at-risk of developing anaemia compared to the others. In the current study, no association was observed between the mean Hb and month neither was there any association between mean Hb and season.

Further studies will therefore be required to ascertain the aetiology and risk factors for anaemia in the study population as well as make prepositions aimed at reversing the current high rate of anaemia in the study population.

## Abbreviations

CI: Confidence interval
Hb: Haemoglobin concentration
OR: Odd ratio
